# Frontal ataxia: historical aspects and clinical definition

**DOI:** 10.1055/s-0043-1775886

**Published:** 2023-10-29

**Authors:** Patrícia Áurea Andreucci Martins Bonilha, Beatriz Cassarotti, Thabata Emanuelle Martins Nunes, Hélio Afonso Ghizoni Teive

**Affiliations:** 1 Universidade Federal do Paraná, Hospital de Clínicas, Departamento de Medicina Interna, Serviço de Neurologia, Unidade de Distúrbios do Movimento, Curitiba PR, Brazil.

**Keywords:** Ataxia, Spinocerebellar Degenerations, Frontal Lobe, Gait Ataxia, Gait Disorders, Neurologic, Ataxia, Degenerações Espinocerebelares, Lobo Frontal, Marcha Atáxica, Transtornos Neurológicos da Marcha

## Abstract

Frontal ataxia, originally described by Bruns, is characterized by the presence of signs of frontal lobe dysfunction, such as perseveration, paratonia, frontal release signs, cognitive changes, and urinary difficulty, associated with imbalance, slow gait, broad-based, the presence of postural instability and falls, retropulsion, and bradykinesia in the lower limbs. The goal of the present study is to recall the historical aspects of this condition, to draw attention to the importance of this clinical finding for the differential diagnosis of ataxias and to review the main semiological differences between primary ataxias (frontal, cerebellar, and sensory ataxia).

## Resumo

A ataxia frontal, originalmente descrita por Bruns, caracteriza-se pela presença de sinais de disfunção do lobo frontal, como perseveração, paratonia, sinais de liberação frontal, alterações cognitivas e dificuldade urinária, associados a desequilíbrio, marcha lenta, base ampla, presença de instabilidade postural e quedas, retropulsão e bradicinesia em membros inferiores. O objetivo do presente trabalho é recordar os aspectos históricos desta condição, ressaltar a importância deste achado clínico para o diagnóstico diferencial das ataxias e revisar as principais diferenças semiológicas entre as ataxias primárias (ataxia frontal, cerebelar e sensitiva).

## INTRODUCTION

The term “ataxia” comes from the Greek word *taxis,* which means “order;” therefore, to denote a disorder of coordination and balance, the word “ataxia” was chosen. 1 2 3 4 Ataxias can be classified as primary or secondary, as well as hereditary or sporadic. Cerebellar ataxia (CA) is a syndrome caused by impairment of the afferent or efferent projections, including several signs and symptoms, such as gait ataxia, dysarthria, nystagmus, tremor, and cognitive dysfunction. 1 2 3 4 5 Afferent or sensory ataxia is due to damage of the proprioceptive pathways and is defined by the presence of gait or limbs ataxia associated with Romberg sign, impairment of joint position and/or vibration senses, and absence of nystagmus and cerebellar dysarthria. 1 2 5 6 There are other types of ataxia, such as vestibular, thalamic, and frontal, and all these types of ataxias are considered controversial entities in the neurological literature. 7 8 With the present review, the authors intent to present diagnostic considerations for frontal ataxia.

## Frontal ataxia

**Figure 1:**
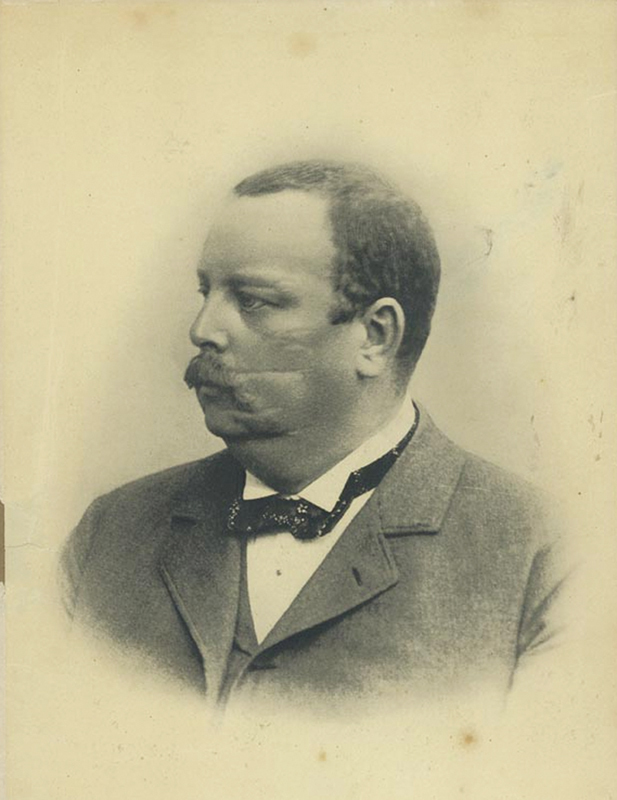
Ludwig Bruns (25 June 1858–9 November 1916). (Reproduced from Google Images, [Wikipedia], July 07, 2023).

Historically, Ludwig Bruns (1858–1916), a neurologist born in Germany, in his 1892 publication, was the first to use the term frontal ataxia (FA), when he described cases of imbalance associated with lesions of the frontal lobe 9 ( Figure. 1 ). In the clinical case described by Bruns, the patient had a frontal lobe tumor. Bruns also became known for the description of the syndrome that bears his name, in 1902, in which there is the presence of headache, vomiting, and sudden attacks of vertigo and syncope. 10 This syndrome is due to an obstruction of the flow of the cerebrospinal fluid during changes of posture of the head. The main causes are cysts or tumors of the third and fourth ventricules. 10 Additionally, he also described the Bruns sign or law, in which there is a complete transverse section of the spinal cord and the reflexes and muscular tone below the level of the lesion are lost. 10 In 1926, Gerstmann and Schilder described two patients with frontal lobe lesions that were not able to walk even supported, then, presenting the term “gait apraxia.” 11 Later, Thompson and Marsden conducted a case series involving patients with arteriosclerotic encephalopathy (Biswanger disease), highlighting a significant difficulty in walking attributed to trunk instability and gait ataxia. 12 In recent years, little attention has been given to the study of the so-called FA. The literature currently available associates FA with gait apraxia, higher level gait disorders, as well as elderly gait syndromes. 13 14 Thompson, in his classic article, lists a series of signs and symptoms suggestive of FA, which are the presence of imbalance, slow, broad-based, and magnetic gait, also with the presence of falls, retropulsion, and frontal signs such as perseveration, hypokinesia, paratonia, frontal release signs, cognitive changes, and urinary difficulty. 13 In general, the differential diagnosis with cerebellar gait disorders and Parkinson's disease is based on the lack of appendicular ataxia, dysarthria, and nystagmus, which are common in cases of cerebellar ataxia, and the absence of resting tremor, facial hypomimia, voluntary movements of the upper limbs, and narrow-based gait expected in Parkinson's disease. 13 The main abnormalities found on neuroimaging exams in patients with FA are periventricular white matter changes, including leukoaraiosis, microangiopathy, and lacunae, in addition to the presence of hydrocephalus. 13 14 15 16 A possible explanation for FA would be the interruptions of the connections between the frontal lobe cortex and subcortical structures, thus including the basal ganglia, the cerebellum, and also the brainstem, all related to gait control. 13 14 15 16 The differential diagnosis with sensory or afferent ataxia can be made by the absence of Romberg's sign, and signs of deep sensitivity dysfunction. Table 1 summarizes the main differences between cerebellar, sensory/afferent, and frontal lobe ataxias.

**Table 1:** Differential diagnosis of cerebellar, sensory, and frontal ataxias

Clinical Features	Cerebellar ataxia	Sensory ataxia	Frontal ataxia
Nystagmus	Present	Absent	Absent
Ocular movement disorders	May be present	Absent	Absent
Dysarthria	Present	Absent	May be present
Limb ataxia	Present	Lower limbs dysmetria	Absent
Trunk ataxia/postural instability	Present	Present in advanced disease	May be present
Gait ataxia	Present	Present	Present
Reflexes	Normal or exacerbated (pendular reflex)	Hyporeflexia or areflexia	Normal
Vibratory sensibility	May be reduced/absent in advanced disease	Reduced/absent	Normal
Proprioception	Normal	Present	Normal
Romberg sign	Absent	Present	Absent
Bradykinesia	Absent	Absent	Present in lower limbs
Frontal signs	Absent	Absent	Present

## DISCUSSION

Frontal ataxia can be characterized by a combination of clinical neurological signs that represent dysfunction of the frontal lobe and its connections with the basal ganglia and dentate nucleus of the cerebellum. 13 Frontal ataxia is thought to be due to dysfunction of the frontopontocerebellar tract (Arnold's Bundle). This tract begins in the frontal cortex, travels through thepontocerebellar peduncle and ends in the cortex of the contralateral cerebellum. 17 Thus, in addition to the classic signs of cognitive dysfunction (mild cognitive disorder or dementia), signs of frontal lobe release (with the presence of palmomental, nasolabial, nasopalpebral, grasping reflexes), the presence of imbalance (dystaxia and gait ataxia), and retropulsion and hypokinesia (predominantly in the lower limbs). 13 As some signs can be confused with those that occur in cerebellar ataxia and Parkinson's disease, it is necessary to define that there is no presence of cerebellar dysarthria, nystagmus, resting tremor, parkinsonian rigidity, in addition to bradykinesia on the face and in the upper limbs. 13 14 Frontal ataxia still generates many discussions in the neurological literature, with the definitions of gait apraxia, “ *marche a petit pas* ,” and the so-called highest-level gait disorders, as defined by Nutt et al. 16 In this group of gait disorders, known as elderly gait syndromes, are the so-called cautious gait, subcortical disequilibrium, frontal disequilibrium, isolated gait ignition failure, and frontal gait disorder. 16 Based on the great importance of the frontal lobe in walk control, we must remind that the presence of clinical features such as broad-base, hypokinetic, magnetic gait, associated with postural instability, with or without cognitive impairment, especially in elderly patients with extensive microangiopathy or hydrocephalus, may raise suspicion of FA, 13 15 16 thus providing a more accurate diagnosis and specific treatment, which may avoid extensive futile investigations, such as a tap-test in normal pressure hydrocephalus suspicion. 15 16
